# A validation study of loci associated with mastitis resistance in two French dairy sheep breeds

**DOI:** 10.1186/s12711-019-0448-8

**Published:** 2019-02-13

**Authors:** Claire Oget, Charlotte Allain, David Portes, Gilles Foucras, Alessandra Stella, Jean-Michel Astruc, Julien Sarry, Gwenola Tosser-Klopp, Rachel Rupp

**Affiliations:** 1 GenPhySE, Université de Toulouse, INRA, ENVT, Castanet-Tolosan, France; 2 INRA, Domaine de La Fage, 12250 Saint-Jean-et-Saint-Paul, France; 3 IHAP, Université de Toulouse, INRA, ENVT, Castanet-Tolosan, France; 4 Parco Tecnologico Padano (PTP), Via Einstein, Cascina Codazza, 26900 Lodi, Italy; 5 Institut de l’Elevage, 31321 Castanet-Tolosan, France

## Abstract

**Background:**

The identification of loci associated with resistance to mastitis or of the causative mutations may be helpful in breeding programs for dairy sheep as it is for cattle worldwide. Seven genomic regions that control milk somatic cell counts, an indirect indicator of udder infection, have already been identified in sheep (Spanish Churra, French Lacaune and Italian Sardinian–Lacaune backcross populations). In this study, we used a 960 custom-designed ovine single nucleotide polymorphism (SNP) chip in Lacaune and Manech Tête Rousse dairy sheep to validate these seven genomic regions associated with mastitis.

**Results:**

The most significant SNP (*rs868996547)* on *Ovis aries* chromosome (OAR) 3 was a previously described mutation in the *suppressor of cytokine signalling 2* (*SOCS2*) gene. An antagonist effect of this causal candidate between health and growth in Lacaune sheep was confirmed. Effects of the mutation on the infectious status of the udder, i.e. increases in milk somatic cell counts and bacteria shedding, were also identified. This SNP was not present in the data available on Manech Tête Rousse. Three other regions associated with mastitis were also confirmed on OAR16 (Manech Tête Rousse), 19 (Lacaune) and 2 (both breeds). For the OAR2 region, we validated previously detected SNPs in several other breeds (Sarda, Churra, and Chios). For significant SNPs in the four mastitis regions, the effect varied from 0.24 to 0.67 phenotypic standard deviation of the traits. Two of the mastitis quantitative trait loci (QTL) regions (OAR2 and 16) that we validated here were also associated in opposite ways with milk production traits in both populations.

**Conclusions:**

These results indicate, at least in part, a genomic basis for the trade-off between milk production and mastitis resistance. Four of the seven mastitis QTL regions that were previously identified in independent populations, were confirmed in this study, which demonstrates partial sharing of mastitis-related genetic mechanisms between different distant dairy sheep populations.

## Background

Mastitis is an inflammation of the mammary gland, which in dairy sheep is mostly due to bacterial infections by *Staphylococci* [1]. Mastitis is a serious burden for the milk industry due to the altered quality of milk and increased cost of flock renewal. Beside hygienic measures, genetic selection for improved resistance to mastitis is now implemented in breeding programs for several breeds of dairy ruminants worldwide [2]. However, its application to dairy sheep is still rare, mainly because the recording cost per animal, relative to potential income, is prohibitive for many traits other than production traits. In sheep, the identification of loci that are associated with resistance to udder infection or the causative mutations may be helpful in selection. However, resistance to mastitis is highly complex and the genetically determined biological basis behind this trait remains unknown.

Several quantitative trait loci (QTL) regions that control milk somatic cell count (SCC), an indirect indicator of udder infection, have been identified in dairy sheep through the EU-funded 3SR project (Sustainable solutions for small ruminants, FP7-KBBE-245140) [3]. For one of these QTL, Rupp et al. [4] identified a single nucleotide polymorphism (SNP) in the coding frame of the *suppressor of cytokine signalling 2* (*SOCS2)* gene as the putative causal mutation associated with high SCC in the Lacaune breed. A few QTL regions were then confirmed by Banos et al. [5] in a population of the Greek Chios breed using four mastitis indicator traits, namely clinical mastitis occurrence, milk SCC, total viable bacterial count in milk and the California mastitis test.

The objective of our study was to confirm the ovine QTL that control mastitis resistance in two independent dairy sheep populations, using a 960 custom-designed ovine SNP chip.

## Methods

Two independent French dairy sheep populations were used: Lacaune ewes (N = 504) from a divergent selection based on extreme breeding values for SCC at the experimental facility of La Fage (INRA, UE 321, Roquefort, France) [6], and Manech Tête Rousse rams (N = 145) raised in the CDEO (Ordiarp, France) testing station in 2013 (birth year from 2008 to 2011). Among the 504 Lacaune individuals, 213 ewes belonged to the high SCC line (42.2%) and 291 to the low SCC line (57.7%). The selection lines were about three genetic standard deviations (SD) apart [6].

In the Lacaune ewe population, milk yield, fat content, protein content, and SCC were measured monthly at morning milking. Test-day SCC were log-transformed for normality into SCS [7]. The arithmetic averages of the first lactation test-days were then computed and corrected for year of sampling for fat content (FAT_L1), protein content (PROTEIN_L1), and SCS (LSCS_L1). The milk yield (MILK_L1) trait was computed as the trait used for genetic evaluation, i.e. the 250-day cumulative production adjusted for lactation length and standardized to an adult production (× 1.3). MILK_L1 was multiplied by 1.3 to follow the definition used in the Manech Tête Rousse breed for genetic evaluation and allow direct comparison of average milk production between both breeds.

*Staphylococcus spp.* abundance in milk was measured at three-time points during the first lactation by a qPCR-based technique developed at the “Interactions Hôtes – Agents Pathogènes” (IHAP) laboratory (Toulouse, France). Briefly, milk was collected aseptically from each half udder independently after precleaning and disinfecting the teat apex using a cotton wool moistened with 70% alcohol. Whole milk was centrifuged (6000*g*; 20 min) before two consecutive enzymatic proteolytic treatments with lysozyme and proteinase K. DNA was extracted using a Biosprint 96 semi-robotic workstation and DNA Blood kits (QIAGEN), and finally eluted in 50 µL of distilled water. An internal DNA control (QIAGEN) was used to assess recovery and lack of qPCR inhibitors. High-throughput qPCR in 384-well format was performed on 1 µL of DNA extract in a total volume of 5 µL using *tuf*-specific primers (tuf 5′-CAC GAC CAG TGA TTG AGA ATA CG and tuf 3′-CCA ATG CCA CAA ACT CGT GA), probe (CCA TTC ATG ATG CCA GTT G), and the Quantifast Pathogen PCR kit (QIAGEN). The proportion of inhibited samples was lower than 5%. Values above the cycle threshold were compared to a standard curve obtained from known amounts of genomic DNA from a *Staphylococcus aureus* laboratory strain and expressed as a bacterial titre (quantity of equivalent bacterial genomes per volume of milk), on a logarithmic scale. The three results were averaged for each ewe and corrected for the effects of month and year of sampling (STAPH_L1).

Chronic mastitis was based on the presence of mammary abscesses, recorded by clinical examination (ABSCESS_L1). Animals were noted as “1” (case) when the presence of at least one abscess was detected at least twice, whereas animals were noted as “0” (control) when they were found to be healthy (without any abscess) at least three times during the first lactation.

Each ewe was weighed at birth (W_BIRTH), at 100 days (W_DAY_100) and 250 days (W_DAY_250), after the first (on average 412 days, W_1ST_LAMB) and second lambing (on average 744 days, W_2ND_LAMB), and at the age of 920 days (W_DAY_920). Phenotypes were corrected for year and feeding method (breastfeeding or artificial suckling). Basic statistics are in Table 1.

**Table 1:** Number of animals with a phenotype (N) and basic statistics for the Lacaune population of 504 ewes

Trait	N	Mean (± SD)
LSCS_L1	494	3.1 ± 1.6
FAT_L1 (g/L)	494	64.1 ± 7.1
PROTEIN_L1 (g/L)	494	53.5 ± 3.4
MILK_L1^a^ (L)	493	334.9 ± 61.9
STAPH_L1	404	0.7 ± 0.8
ABSCESS_L1	376	15 cases/361 controls
W_BIRTH (kg)	502	3.9 ± 0.6
W_DAY_100 (kg)	492	28.7 ± 3.0
W_DAY_250 (kg)	472	50.6 ± 4.1
W_1ST_LAMB (kg)	438	64.7 ± 6.7
W_2ND_LAMB (kg)	393	73.4 ± 7.7
W_DAY_920 (kg)	378	67.3 ± 7.2

^a^MILK_L1 is the 250-day cumulative production adjusted for lactation length and standardized to an adult production (× 1.3)

In the Manech Tête Rousse population, SCC and milk production traits were obtained from the official milk records. In this breed, milk yield is measured monthly and SCC, fat and protein contents are measured three to four times during the first three lactations [8]. For association mapping, we used the daughter yield deviations (DYD) [9] from regular national genetic evaluations for milk production traits (MILK, PROTEIN, and FAT) and lactation average somatic cell scores (LSCS). DYD correspond to the average performance of the daughters of a ram, corrected for the environmental effects and the genetic value of the dams (Table 2).

**Table 2:** Number of animals with a phenotype (N) and basic statistics for the Manech Tête Rousse population of 145 rams and their 15,722 offspring

Trait	Rams^a^		Offspring ewes^b^	
	N	Mean (± SD)	N	Mean (± SD)
LSCS	145	0.1 ± 0.6	15,115	3.6 ± 1.5
FAT (g/L)	145	0.9 ± 4.2	15,368	61.9 ± 8.4
PROTEIN (g/L)	145	0.2 ± 2.6	15,368	49.3 ± 4.4
MILK^c^ (L)	145	84.7 ± 25.2	15,722	270 ± 79

^a^Phenotypes are DYD expressed as deviations from the average population used for genetic evaluation

^b^Phenotypes are the observed performances during the first lactation

^c^MILK is the 250-day cumulative production adjusted for lactation length and standardized to an adult production (× 1.3)

Both Lacaune and Manech Tête Rousse populations were genotyped with a 960 custom-designed ovine SNP chip [10]. The chip was designed and developed within the 3SR EU project [3] based on several QTL for SCC that were previously identified in Spanish Churra [11], French Lacaune [4] and Italian Sardinian–Lacaune backcross populations ([12] and personal communication). Using these previous association studies, seven regions of interest (Table 3) on *Ovis aries* (OAR) chromosomes 2, 3, 5, 16 and 18 were selected based on commonalities among populations found at the time (in 2012) or on their high significance. SNPs were selected within these regions from the 54 K or 800 K Illumina ovine chips [13] or from novel genome sequencing within the 3SR project. The 10 SNPs in the OAR3 region included the causal mutation in the *SOCS2* gene and nine other closely linked loci that had been identified by Rupp et al. [4]. Genomic positions refer to the ovine reference genome v3.1 [14]. After quality control, the following SNPs were excluded from the analyses: non-polymorphic SNPs, SNPs with a missingness rate higher than 5%, with a minor allele frequency lower than 2%, and SNPs that deviated from Hardy–Weinberg proportion (*p* < 1E−05), thus, 745 and 708 SNPs were selected for the Lacaune and Manech Tête Rousse populations, respectively.

**Table 3:** SNPs of the 3SR-mastitis-960-SNP custom chip, selected within the 3SR EU project for their association with mastitis-related traits

OAR	Discovery population	QTL^a^ (Mb)	Interval (Mb)		Origin of the SNP			Number of SNPs		
			Start	End	54 K chip	800 K chip	SEQ	Before QC	After QC LAC	After QC MTR
2	LAC	125	124.6	125.5	19	10		29	24	24
2	SAR	206.7	204.2	209.7	106	146		252	196	180
	CHU	208.7								
3	LAC	129.7	129.7	130.1	4	6	10	20	13	12
5	LAC	96.5	95.8	96.2	11	9		20	17	16
12	CHU	18.3	17.9	20.4	54	76		130	101	97
	SAR	18.4								
16	LAC	36.2	28.3	36.8	144	110		254	202	183
19	CHU	26.3	23.6	28.9	75	180		255	202	196
	LAC	28.6								

*OAR Ovis aries* chromosome, *LAC* Lacaune, *SAR* Sardinian–Lacaune backcross, *CHU* Churra, *QTL* quantitative trait locus, *Mb* megabase, *SEQ* sequence, *QC* quality control, *MTR* Manech Tête Rousse

^a^QTL location

Genome-wide association studies (GWAS) were performed for each phenotype using the polygenic univariate mixed model approach implemented in the genome-wide efficient mixed-model association (GEMMA) software [15]. The polygenic effect was fitted using a covariance structure according to the genomic relationship matrix. Corrections were applied to account for multiple testing. First, a Bonferroni correction of α = 5% was applied (significance threshold = α/number of SNPs). SNPs with a *p* value less than 6.6E−05 and less than 7.1E−05 were considered as highly significantly associated for the Lacaune and the Manech Tête Rousse populations, respectively. Since association tests are not independent, due to the large number of SNPs in high linkage disequilibrium within the QTL regions, and to several traits being highly correlated, a less restrictive suggestive significance threshold was also calculated (significance threshold = [α/number of independent regions in the chip]/number of independent variables in the study). Table 3 lists the seven independent regions that were found to contain such SNPs on the 960 custom-designed ovine SNP chip. We used two methods to obtain the number of independent variables: a factor analysis of mixed data (FAMD) for the Lacaune population, for which we had to consider quantitative (LSCS_L1, FAT_L1, PROTEIN_L1, MILK_L1, STAPH_L1, weights) and qualitative (ABSCESS_L1) phenotypes, and a principal component analysis (PCA) for the Manech Tête Rousse population, for which all phenotypes were quantitative. FAMD and PCA using phenotypes were computed using the FactoMineR package [16] of the R software [17]. According to the clustering elbow method, the number of independent variables is the marginal point where the percentage of variance explained by the PCA dimensions drops and produces an angle in the histogram. This method led us to choose the first five dimensions of the FAMD (Lacaune) and the first two dimensions of the PCA (Manech Tête Rousse), which explained 77.5% (Lacaune) and 79.6% (Manech Tête Rousse) of the variance. Therefore, we used N1 = 5 (Lacaune) and N2 = 2 (Manech Tête Rousse), the number of independent variables in the study, leading to suggestive significance thresholds of 1.4E−03 and 3.6E−03, respectively.

## Results and discussion

Significant SNPs from the GWAS are in Table 4 (Lacaune) and Table 5 (Manech Tête Rousse). The first noteworthy result concerns the highly significant region on OAR3 in the Lacaune population. Indeed, three SNPs, which were associated with mastitis and growth traits, were detected at the Bonferroni threshold. The most significant SNP (*rs868996547*, *p* value = 3.0E−07) was the causal mutation in the *SOCS2* gene, previously reported by Rupp et al. [4]. This mutation causes a loss in functional activity of the SOCS2 protein, which is involved in inflammatory response control and growth [18] through the JAK/STAT/SOCS pathway. The lowest *p* values and highest estimates of effects for this SNP were observed for both mastitis traits (LSCS_L1 and STAPH_L1) and four of the six weight traits. Corresponding effects varied from 0.33 SD for W_DAY_100 to 0.50 SD for LSCS_L1. Thus, we confirmed an adverse effect of the *SOCS2* gene point mutation on mammary inflammation and growth, as reported by Rupp et al. [4]. We also found that the mutation had an unfavourable effect on the infectious status of the udder (0.38 SD), since the low-frequency allele increased cell counts and bacteria shedding in milk. All these results confirm the pleiotropic effect of the *SOCS2* mutation on body growth and the host’s control of mastitis. This SNP did not segregate in the Manech Tête Rousse population although 12 other SNPs segregated in this narrow genomic region (Table 3). No QTL for mastitis was detected in this region for the Manech Tête Rousse population (Table 5), which provides further evidence that *rs868996547* is a strong candidate in Lacaune but is absent from Manech Tête Rousse. Moreover, we found that there was no effect of the SNPs of the same region on OAR3 when the SNP considered as causal (*rs868996547*) was included as a fixed factor in the model for the Lacaune population analyses. Indeed, we observed an increase of the *p* values of the SNPs that surround the mutation for all traits for which the association was previously significant. For example, the minimum *p* value for the LSCS_L1 trait in the OAR3 region was 1.5E−02 (*rs425616833*), which confirmed that the other SNPs in the region do not explain any additional variance.

**Table 4:** List of SNPs associated with the different phenotypes for the Lacaune population genotyped with the 3SR-mastitis-960-SNP custom chip

OAR	SNP ID	SNP position	MAF %	Estimation of SNP effect	SE	*p* value of Wald test	Significant	Trait
2	rs414463145	204256340	23	− 2.1	0.6	1.1E−03		FAT_L1
	rs420100221	204333224	32	− 2.0	0.6	6.9E−04		FAT_L1
	rs409223720	206118959	46	0.0	0.0	1.3E−03		ABSCESS_L1
	rs406554330	206311785	35	0.1	0.0	8.2E−04		ABSCESS_L1
	rs421656393	207088920	34	2.0	0.6	1.2E−03		FAT_L1
	rs409595486	209135680	12	2.6	0.8	7.6E−04		FAT_L1
3	*rs428428896*	*129680367*	22	*0.6*	*0.1*	*9.6E*−*06*	***	*LSCS_L1*
			22	0.3	0.1	1.4E−03		STAPH_L1
			21	2.0	0.6	1.1E−03		W_1ST_LAMB
	rs425616833	129685397	39	− 0.4	0.1	3.2E−04		LSCS_L1
	*rs868996547*	*129722200*	17	*0.8*	*0.1*	*3.0E*−*07*	***	*LSCS_L1*
			17	0.3	0.1	6.8E−04		STAPH_L1
			17	1.0	0.3	6.0E−04		W_DAY_100
			17	1.5	0.4	3.7E−04		W_DAY_250
			16	2.4	0.7	5.0E−04		W_1ST_LAMB
			15	2.8	0.9	9.5E−04		W_DAY_920
	*rs426941860*	*129927538*	20	*0.7*	*0.1*	*5.2E*−*07*	***	*LSCS_L1*
			20	0.8	0.3	1.2E−03		W_DAY_100
			19	2.4	0.7	1.0E−03		W_DAY_920
16	rs404369966	28321220	25	− 0.2	0.0	3.2E−04		W_BIRTH
	*rs403769730*	*35801129*	6	*34.5*	*8.4*	*5.2E*−*05*	***	*MILK_L1*
			6	1.5	0.4	3.4E−04		W_DAY_100
			7	2.1	0.6	6.8E−04		W_DAY_250
19	rs412825949	25639818	32	− 0.3	0.1	4.1E−04		STAPH_L1
	*rs416350585*	*25684229*	14	*3.6*	*0.9*	*3.4E*−*05*	***	*W_DAY_920*
	rs400011699	28086791	38	− 14.9	4.0	2.6E−04		MILK_L1

SNPs in italic are significant with the Bonferroni threshold and the others are significant with the suggestive threshold

*OAR Ovis aries* chromosome, *MAF* minor allele frequency, *SE* standard error of the estimated SNP effect

**Table 5:** List of SNPs associated with the different phenotypes for the Manech Tête Rousse population genotyped with the 3SR-mastitis-960-SNP custom chip

OAR	SNP ID	SNP position	MAF %	Estimation of SNP effect	SE	*p* value of Wald test	Trait
2	rs403180214	206231638	50	− 0.3	0.1	1.7E−04	LSCS
	rs430641657	207218842	12	16.0	4.3	2.9E−04	MILK
	rs425558509	208650955	42	− 1.5	0.5	2.6E−03	FAT
	rs398918249	209409342	43	− 1.0	0.3	1.3E−03	PROTEIN
16	rs404369966	28321220	26	1.0	0.3	3.6E−03	PROTEIN
			26	− 10.2	3.4	2.7E−03	MILK
	rs428277629	28526130	35	1.0	0.3	2.0E−03	PROTEIN
	rs418639132	28632456	41	1.0	0.3	1.0E−03	PROTEIN
	rs405638287	29030021	37	1.1	0.3	7.4E−04	PROTEIN
	rs399031726	29072599	36	1.2	0.3	2.5E−04	PROTEIN
	rs422927329	29304504	37	1.2	0.3	1.1E−04	PROTEIN
	rs402269497	30319608	19	− 12.5	3.8	1.2E−03	MILK
	rs421638047	30692759	28	1.9	0.6	1.1E−03	FAT
			28	1.2	0.3	6.1E−04	PROTEIN
			28	− 12.9	3.4	2.1E−04	MILK
	rs409981325	31298560	10	0.4	0.1	1.9E−03	LSCS
	rs409954614	34424863	37	1.0	0.3	1.2E−03	PROTEIN

There are no significant SNPs at the Bonferroni threshold for this breed, all the SNPs presented in this table are significant with the suggestive threshold

*OAR Ovis aries* chromosome number, *MAF* minor allele frequency, *SE* standard error of the estimated SNP effect

Then, we applied suggestive thresholds, which allowed us to confirm three other regions that are associated with mastitis. In the Lacaune population, regions on OAR2 and 19 are associated with the ABSCESS_L1 and STAPH_L1 traits, respectively, and regions on OAR2 and 16 are also significant for the LSCS trait in the Manech Tête Rousse population. For significant SNPs, the effect varied from 0.38 SD (OAR19 in Lacaune) to 0.67 SD (OAR16 in Manech Tête Rousse) of the traits (Tables 4, 5). These three QTL regions had already been identified in Sarda (OAR2), Churra (OAR19 and 2) (Table 3) and Chios breeds (OAR2, 16 and 19) [5]. Thus, for these regions, and especially OAR2, our data reinforce the hypothesis of true mastitis QTL, which might involve similar genes and pathways across breeds. Banos et al. [5] suggested several candidate genes for OAR2: *cytotoxic T*-*lymphocyte*-*associated protein 4* (*CTLA4*2; 204,777,523–204,784,522 bp), *inducible T*-*cell co*-*stimulator (ICOS*; 204,851,429–204,873,693 bp), and *isocitrate dehydrogenase 1 (NADP*+*), soluble* (*IDH1*; 209,236,699–209,259,307 bp), which are involved in T-cell responses or mitigation of oxidative damage. However, the region between 204.2 and 209.7 Mbp on OAR2 includes more than 90 genes, which makes the identification of appropriate candidates difficult.

Interestingly, two of the mastitis QTL regions (OAR2 and 16) that were confirmed in the present study, were also associated with milk production traits in both populations. Moreover, the region on OAR16 is strongly (Bonferroni threshold) associated with MILK_L1 (effect = 0.56 SD) in the Lacaune population and with MILK, FAT, and PROTEIN (effect = 0.50 SD, 0.45 SD and 0.46 SD, respectively) in the Manech Tête Rousse population. Thus, the underlying QTL could be a QTL for milk production that has an indirect impact on mastitis. In Lacaune, the positive sign of the estimated effects of SNP *rs403769730* shows that this QTL is favourable for milk production (Table 4), i.e. leading to an increase in milk quantity, but unfavourable for LSCS_L1 (results not shown), i.e. leading to an increase in somatic cell count. A similar pattern is observed for SNP *rs421638047* in Manech Tête Rousse, for which the estimated effects have a negative sign, i.e. leading to a decrease in MILK which is unfavourable for milk yield (Table 5) and a decrease in LSCS which is favourable for the health of the animal (results not shown). These results are in agreement with the positive and antagonistic correlation that exists between mastitis and milk production trait in Lacaune [19] and other ovine [20] and bovine breeds [21], which indicates, at least in part, a genomic basis for the trade-off between milk production and mastitis resistance.

## Conclusions

We confirmed four out of seven QTL regions for mastitis in the Lacaune population, and only two in the Manech Tête Rousse population. This is consistent with the fact that Lacaune belongs to the breeds for which these regions were first discovered, although the individuals were different. The two significant regions detected in the Manech Tête Rousse population are rather encouraging, unlike a similar study on nematode resistance where QTL validation was inconclusive [22]. These results demonstrate that mastitis-related genetic mechanisms are shared between different distant dairy sheep populations.
